# American Medical Association

**Published:** 1890-06

**Authors:** 


### EDITORIAL.

### American Medical Association.

The annual meeting of this body was held in Nashville, beginning May 20th, and continuing four days.

It was a very successful meeting, there was a large number in attendance and much excellent work accomplished. It would be altogether impracticable to attempt here to give a synopsis even of what was done.

The work done by the various sections of the association was very satisfactory indeed. Of course we know more, personally, of that which was done in the dental section than in the others. Notwithstanding, most flattering reports were made from all the sections. There was quite a number of papers read in this section by medical men; papers of great interest which were fully discussed.

The paper on "The Relation of Tropho-Neurosis to Diseases of the Mouth and Jaws with Special Reference to Syphilitic Neurosis," by Dr. G. F. Lydston, of Chicago, was a most excellent paper and dealt very thoroughly with the science of this subject.

Dr. Lydston has made special study of this subject, and has had a very extensive clinical experience, so that what he says upon the subject may well be regarded as authority.

The paper by Dr. H. O. Marcey, of Boston, on Cure for Cleft Palate by a Double Operation and Closure with a Buried Tendon Suture, was one of rare interest. An original and entirely successful operation was described in this paper, in which, after the operation, the parts being rendered fully antiseptic, were absolutely secured against any septic influences to the parts, and maintained in absolute quiet.

A paper on The Value of Illustrations in the Lecture Room, by Dr. McIntosh, was a very instructive and interesting one. The doctor presented several points upon this subject that we do not remember elsewhere to have seen.

A paper was presented by Dr. Thos. H. Manley, of New

York, on Early Operations in Congenital Clefts. In this paper Dr. Manley advocated the operation for congenital clefts at an earlier period than we have ever known suggested before. He strongly recommends this operation within three or four days after birth. The reasons for this are, that it is more easily performed than later in life; that the tissues unite more readily; that inflammation and suppuration are not so likely to occur and that the case subsequent to the operation is more easily controlled. The cases Dr. Manley gave in illustration certainly well bear out his theory.

A most excellent paper on Tumors and Growths of the Mouth and Jaws, was presented by Dr. Hamilton, of Columbus, Ohio. This was also a very instructive and interesting paper.

The authors of each of these papers had ample opportunity and sustained the positions they had taken by explanations, called for in a wide range of questioning by the members present.

The work done in this section was quite different, in many respects, from that done in the ordinary dental societies. It dealt largely with principles, and the intimate relation between principles and practice was clearly brought out. There was very little of merely practical dental matters. There was no time wasted in merely business matters; not over fifteen to twenty minutes were devoted to any thing of this kind during all the sessions of the section; not over ten minutes were occupied in the election of officers; and we think every one present must have been thoroughly convinced of the value and importance of a dental section in connection with the American Medical Association.

The next meeting of the association will be held in Washington City, when, we have no doubt, there will be a much larger attendance on this section than ever before.

